# Perspectives and practices of ototoxicity monitoring

**DOI:** 10.4102/sajcd.v67i1.685

**Published:** 2020-05-19

**Authors:** Jessica Paken, Cyril D. Govender, Mershen Pillay, Vikash Sewram

**Affiliations:** 1 Discipline of Audiology, School of Health Sciences, University of KwaZulu-Natal, Durban, South Africa; 2 African Cancer Institute, Department of Global Health, Faculty of Medicine and Health Sciences, Stellenbosch University, Cape Town, South Africa

## Abstract

**Background:**

Treatment of cancer with cisplatin can result in hearing loss. Given the increasing burden of cancer in Africa, appropriate and timely identification, intervention and management of hearing loss in affected patients is of paramount importance.

**Objectives:**

This study describes the perspectives and practices of healthcare professionals in relation to cisplatin-associated ototoxicity at an institution treating patients diagnosed with cancer.

**Method:**

A concurrent triangulation study design was used to collect quantitative data from seven oncologists, nine nurses and 13 pharmacists using self-administered questionnaires, and qualitative data from four audiologists through semi-structured interviews for this hospital-based study, conducted in South Africa.

**Results:**

Levels of awareness of cisplatin-associated ototoxicity varied with only 33% of the nursing personnel being aware in comparison to the oncologists and pharmacists. Oncologists were identified as the main custodians for providing information to patients. Whilst 82% of the participants considered the audiologist to be part of the oncology team, there was no provision for ototoxicity monitoring in the chemotherapy protocols, nor any ototoxicity-monitoring programme in place. There was no evidence that knowledge of cisplatin-associated ototoxicity translated into an appropriate management strategy for such patients.

**Conclusion:**

Healthcare personnel overseeing the care and management of cancer patients need to improve their awareness of ototoxicity and refer timeously for audiological evaluation. Audiologists require greater awareness of monitoring programmes to appropriately implement and manage such programmes within a cancer platform and be part of a multidisciplinary team.

**Keywords:**

awareness; cisplatin; South Africa; ototoxicity; cervical cancer; healthcare personnel.

## Introduction

South Africa is experiencing an increase in the overall burden of disease attributable to cancer, with the number of new cancer cases predicted to increase by 46% by 2030 (Ferlay et al., 2013). In light of the country’s quadruple burden of disease, this poses a challenge to the medical fraternity, as many human immunodeficiency virus** (**HIV), tuberculosis (TB) and cancer therapeutics are ototoxic, causing hearing loss from temporary or permanent inner ear dysfunction owing to treatment with such agents (Yorgason, Fayad, & Kalinec, 2006). Furthermore, HIV and TB often occur together, in addition to many acquired immunodeficiency syndrome–related malignancies, such as cervical cancer, Kaposi’s sarcoma and so on, thus compounding the impact of ototoxicity as a result of the combined regimens that are prescribed. Therefore, it is crucial that hearing assessments form part of the holistic management of patients diagnosed with these conditions. However, the public health importance of ototoxicity is often unheeded, to the detriment of affected patients. This negatively affects their communication ability, often resulting in educational and economic shortcomings, and social isolation (Paken, Govender, Pillay, & Sewram, 2016). In limited resource environments, such outcomes are often intensified because of the lack of rehabilitation and social services.

Whilst local statistics are highly underrepresented, the World Health Organization’s (WHO) global estimate for disabling hearing impairment, defined as more than 40 dB HL impairment in adults and more than 30 dB hearing threshold in children, has more than doubled from 120 million people in 1995 to at least 278 million in 2005. Of these individuals, approximately 80% of the affected population are living in developing countries (Olusanya & Newton, 2007). The challenge in addressing this burden lies not only in early identification of high-risk groups but also in the provision of early interventions. Increasing global estimates for disabling hearing impairment result not only from more people living longer but also external factors such as recreational and occupational noise exposure as well as increased use of ototoxic medications.

The ototoxic effects of cisplatin, such as bilateral high-frequency sensorineural hearing loss and tinnitus (Daldal, Odabasi, & Serbetcioglu, 2007), are compounded in cases where the affected individuals diagnosed with cancer are also HIV-positive and undergoing antiretroviral therapy (ART). They face a double-barrelled effect of ototoxicity due to the ototoxic effects of cisplatin and ARTs (Bisht & Bist, 2011). Cisplatin-associated ototoxicity has been reported by numerous researchers over the years, and it is therefore critical that healthcare professionals understand the impact of this ‘invisible condition’ (Tye-Murray, 2014), as ototoxicity further impacts on the patient’s quality of life. Therefore, to mitigate further deterioration of quality of life, operational processes need to be in place so that impairments that may subsequently arise, including ototoxicity, can be addressed through appropriate and immediate management measures. Hence, one has to ensure operational processes that will minimise the resulting comorbidities from the use of such drug regimens (Paken et al., 2016).

With regard to ototoxicity, an ototoxicity-monitoring programme can avert, to a large extent, the impact of hearing loss. It enables patients undergoing treatment with known ototoxic drugs to be identified early, counselled, monitored and managed through appropriate interventions in a logical, systematic and coherent manner. Such a programme can involve a healthcare team, using evidence-based practices (Schellack, Wium, Ehlert, Van Aswegen, & Gous, 2015), to ensure effective sustainability of such a programme, if implemented, with the patient being the central focus. The team should consist of the oncology nurse, oncologist, audiologist and pharmacist.

Apart from hearing loss, tinnitus and vestibular dysfunction are also by-products of ototoxicity; therefore, sensitivity to a patient’s comorbidity and a resultant comprehensive team approach is crucial to improving the patient’s overall quality of life. Awareness of the adverse effects of cisplatin amongst healthcare personnel involved in the management of affected individuals is of paramount importance to ensure appropriate counselling of the patient and provision of appropriate professional referrals for holistic management.

A team approach is possible only if the team members are knowledgeable of the ototoxic effects of medication, its related symptoms as well as the responsibilities of each of the members within an ototoxicity-monitoring programme. Key responsibilities of the multidisciplinary team involved in ototoxicity monitoring are highlighted in Table 1.

**TABLE 1:** Key responsibilities of the healthcare personnel involved in ototoxicity monitoring of patients receiving cancer chemotherapy.

Healthcare personnel	Responsibilities include
**Audiologists**	Identifying an ototoxic hearing loss Informing the oncologist of such a development Counselling the patient and their family Prescribing amplification devices, such as hearing aids and cochlear implants (American Academy of Audiology, 2009)
**Oncologists (registrars and oncologists)**	Assessing patients for comorbidities and requesting for baseline assessments (Health Professions Council of South Africa) Adjusting the chemotherapy regimen to reduce or prevent further deterioration of hearing (American Academy of Audiology, 2009) Counselling patients on the side effects of cisplatin, including ototoxicity, in an attempt to prepare them for treatment outcomes and help them set realistic expectations (Dabrowski & Hussain-Said, 2010)
**Oncology nurses**	Counselling patients on the side effects of cisplatin, including ototoxicity (Dabrowski & Hussain-Said, 2010) Monitoring ototoxic signs and symptoms and referring when appropriate (Health Professions Council of South Africa, 2018)
**Pharmacists**	Alerting the oncologists and audiologists to those patients who are on other ototoxic medication and therefore at a greater risk for cisplatin ototoxicity.

Whilst there is one international study (Steffens et al., 2014) and three South African studies that have focussed on the knowledge and/or practices regarding ototoxicity (De Andrade, Khoza-Shangase, & Hajat, 2009; Khoza-Shangase & Jina, 2013; Wium & Gerber, 2016), all four studies have focussed on the oncologists and general practitioners. Unfortunately, there are no studies reporting on the variation in practise and awareness of other healthcare professionals such as the nurses, audiologists and pharmacists in relation to cisplatin-associated ototoxicity within a single facility treating cancer patients. It is important to assess awareness and management practices within such a facility, as it provides scope as an ideal environment, if required, for the implementation of an ototoxicity-monitoring programme. This can maximise patient outcomes within the confines of facility-specific infrastructure and resources. It is therefore essential to determine the awareness of healthcare personnel regarding cisplatin-associated ototoxicity and to determine if this awareness, or lack thereof, influences their practice. Even though the ototoxic side effect of cisplatin in the literature, most oncologists do not anticipate or look for ototoxicity in patients receiving cisplatin (Malhotra, 2009). Therefore, the aim of this concurrent mixed methods study was to describe the healthcare professionals’ perspectives of, and current practices for, monitoring ototoxicity in cancer patients receiving cisplatin chemotherapy. The definition of ‘perspectives’ used consistently in the current study refers to participants’ reported knowledge of a situation or fact, whilst ‘practice’ refers to their current management of the affected patient. A greater understanding of the current management practices of affected patients will form a basis for the formulation of a more robust and successful implementation of an ototoxicity-monitoring programme relevant to the South African public health sector.

## Research method and design

### Study design

The study utilised a concurrent triangulation approach, as both quantitative and qualitative data were collected simultaneously from different data sources, and the two databases were used to describe the healthcare professionals’ perspectives of, and current practices for, monitoring ototoxicity in cancer patients receiving cisplatin chemotherapy (Cresswell, 2009). Therefore, whilst the quantitative and qualitative data were collected concurrently and analysed separately, mixing of the data occurred in the results and discussion of this article.

### Setting

The study was conducted at a tertiary hospital in KwaZulu-Natal, South Africa, which is one of the treatment centres for patients with cancer in the province.

### Study population and sampling strategy

Maximal variation sampling was used in the study, as the researcher purposefully sampled individuals who differed on some characteristic, that is, profession (Cresswell, 2012). Healthcare personnel directly involved in the management of patients with cancer, that is, oncologists, oncology nurses and pharmacists as well as audiologists were invited to participate. Physicians, pulmonologists and general nurses were not invited to participate as the study was confined to the oncology unit or clinic, as this was the setting where the greatest influence in terms of appropriate management and referrals of patients with cancer was envisaged to be made.

### Data collection

Qualitative data were collected from individual face-to-face interviews conducted for approximately 30 minutes and audio recorded with the resident audiologists, using inductive inquiry and a semi-structured interview schedule (with predetermined themes), the questions of which are reflected in Table 2. Quantitative data were collected from consenting oncologists, nurses and pharmacists on completion of a 10-minute questionnaire specific to their role in ototoxicity monitoring, as indicated in Tables 3 and 4. The questionnaire for the oncology clinic personnel was adapted from that of De Andrade et al. (2009).

**TABLE 2:** Questions in the semi-structured interview schedule.

Number	Question
1.	How many years have you been practicing audiology?
2.	Describe your client base.
3.	Are you aware of drugs that may result in hearing loss? If yes, list some of the classes of drugs that may lead to this problem.
4.	Describe the auditory complaints that patients on chemotherapy may complain of.
5.	Discuss your role as part of the team that deals with patients with cancer.
6.	What do you think are the key elements of an ototoxicity-monitoring programme?
7.	Do you see a role for audiologists in the above programme? If yes, discuss your role.

**TABLE 3:** Clinic personnel responses to the questionnaire.

Variable	Clinic personnel			
	Oncologists (*n* = 7)		Nurses (*n* = 9)	
	*n*	%	*n*	%
**Experience working with cancer patients (months)**				
< 12	0	0	5	56
12–48	3	42.9	2	22
49–84	3	42.9	2	22
> 84	1	14.3	0	0
**Do the patients, on cancer chemotherapy, complain of any auditory symptoms?**				
Yes	5	71.4	7	77.8
No	2	28.6	2	22.2
**Type of auditory complaints**				
Reduced hearing sensitivity	4	57.1	4	44.4
Pain in the ears	3	42.9	6	66.7
Noise in the ears	2	28.6	0	-
Hypersensitivity to sounds	0	-	0	-
**Who is the patient referred to if there are complaints of reduced hearing sensitivity?**				
Family doctor	0	-	0	-
ENT specialist	3	42.8	8	88.9
Speech language pathologist	0	-	0	-
Audiologist	6	85.7	3	33.3
**Are you aware that certain chemotherapy drugs, for cancer, may cause hearing loss?**				
Yes	7	100	3	33.3
No	0	-	6	66.6
**Do you give patients information about the possible ototoxic effects of medication prior to commencing chemotherapy**				
Yes	6	85.7	7	77.8
No	1	14.3	2	22.2
**Do you provide patients with any recommendations regarding their hearing**				
Yes	3	42.9	5	55.6
No	4	57.1	4	44.4
**Do you consider the audiologist to be part of the team that deals with patients with cancer?**				
Yes	7	100	7	77.8
No	0	-	2	22.2
**Whose responsibility is it to provide patients with information about possible ototoxic effects of medication?**				
Nurses	2	28.6	2	22.2
Oncologists	7	100	9	100
Pharmacists	1	14.3	2	22.2
Audiologists	1	14.3	0	-
**Do you enquire about patient’s history of hearing difficulties?**				
Yes	5	71.4	4	44.4
No	2	28.6	5	55.6
**Do you enquire about patient’s family history of hearing loss?**				
Yes	1	14.3	3	33.3
No	6	85.7	6	66.7
**Do you ask about patient’s medical history and drugs used to treat these conditions?**				
Tuberculosis	7	100	2	22.2
HIV	7	100	5	55.6
Malaria	1	14.3	1	11.1
Pain and fever	5	71.4	5	55.6
**Does your environment have an ototoxicity-monitoring programme?**				
Yes	0	-	0	-
No	5	71.4	1	11.1
Unsure	2	28.6	8	88.9
**Is there a protocol in your environment that indicates when a patient’s hearing should be monitored?**				
Yes	0	-	0	-
No	4	57.1	2	22.2
Not sure	3	42.9	7	77.8

HIV, human immunodeficiency virus; ENT, Ear-Nose-Throat specialist.

**TABLE 4:** Pharmacist’s responses to the questionnaire.

Questions to pharmacists	Responses (*n* = 13)	
	*n*	%
**Do you dispense medication to patients on cancer chemotherapy?**		
Yes	13	100
No	0	0
**How many times in the last month have you been consulted about drug interactions?**		
< 10	13	100
10+	0	0
**Does the hospital have a system that would allow you to identify patients at risk for a hearing loss?**		
Yes	2	15.4
No	11	84.6
**Are you aware that certain chemotherapy drugs, for cancer, may cause hearing loss?**		
Yes	13	100
No	0	0
**Do you alert oncology staff to patients who are at risk for an ototoxic hearing loss?**		
Yes	1	7.7
No	12	92.3
**Do you consider the audiologist to be a part of the team that deals with patients with cancer?**		
Yes	9	69.2
No	4	30.8
**Whose responsibility do you think it is to provide patients with information about the possible ototoxic effects of medication?**		
Nurses	5	38.5
Oncologists	12	92.3
Pharmacists	11	84.6
Audiologists	5	38.5
**Whose responsibility do you think it is to refer patients at risk for a hearing loss to the ototoxicity-monitoring programme?**		
Nurses	2	15.4
Oncologists	13	100
Pharmacists	3	23.1
Audiologists	1	7.7

The questionnaires and interviews aimed to solicit information on perceived awareness of chemotherapy-associated ototoxicity, patient-reported symptoms and perceived roles and responsibilities of the different healthcare professionals within an ototoxicity-monitoring programme as well as their current practices in the identification and management of patients at risk for cisplatin-associated ototoxicity. Interviews were conducted with the audiologists to enable the acquisition of in-depth information, as audiologists are considered the key role players in setting up and implementing an ototoxicity-monitoring programme.

### Data analysis

Following verbatim transcription of the interviews, the transcripts were uploaded for analysis using QSR International’s NVIVO 12 software, and analysed using thematic analysis (Richards, 1999). The main themes were predetermined, whilst the subthemes emerged during analysis of the transcriptions.

Descriptive analysis of the quantitative data included percentage counts. The Fisher’s exact test was used to statistically compare the responses between the oncologists and oncology nurses, and reported where significant differences were observed. The Point-Biserial correlation analysis was used to determine the relationship between the months of clinical experience of the clinical personnel and awareness that certain chemotherapy drugs can cause hearing loss, as well as enquiries with respect of patient history. A *p*-value of <0.05 was considered statistically significant. The qualitative and quantitative data were then triangulated, as there is evidence from different individuals, different types of data as well as different methods of data collection (Cresswell, 2012). This afforded the researchers the opportunity to ensure that the responses were concordant.

### Reliability and validity

A pilot study was conducted with one oncologist, one nurse and one pharmacist who were not included in the main study, prior to data collection. The purpose of the pilot study was to assess any potential problems with the questionnaires and to ensure reliability of the study. No changes were made to the questionnaires based on the results of the pilot study. A pilot study was not conducted with the audiologists because of the limited number of audiologists at the study site. Member checking with the audiologists was used to verify accurate transcription of interviews.

### Ethical consideration

The study was approved by the University Research and Ethics Committee, the Department of Health and the hospital management ([BREC], Ref. No.: BE064/13). All participants were briefed about the nature of the study, the study procedures, their right to withdraw at any time and procedures to maintain confidentiality. They were also informed that their participation was voluntary. Written informed consent was obtained from all participants.

## Results

Thirty-three healthcare personnel, including seven oncologists, nine nurses and 13 pharmacists, all of whom are involved in the management and care of cancer patients, as well as four audiologists (A, B, C and D), participated in the study. All oncologists had more than 1 year of experience managing patients with cancer. The oncologists included registrars and oncologists, with four (57%) having more than 4 years of experience. Five nurses (56%) had less than 1 year of experience managing patients with cancer, whilst two (22%) had more than 4 years of experience. A comprehensive list of the responses by oncologists and nurses is provided in Table 3, whilst that of pharmacists is provided in Table 4.

**FIGURE 1:**
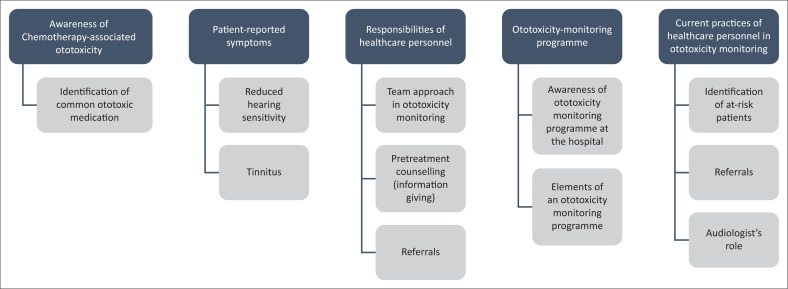
Hierarchy of themes arising from transcripts.

During the interviews, the five major themes included awareness of chemotherapy-associated ototoxicity, patient-reported symptoms and the perceived roles and responsibilities of the different healthcare professionals within an ototoxicity-monitoring programme as well as their current practices in the identification and management of patients at risk for cisplatin-associated ototoxicity. The themes and subthemes are reflected in Figure 1.

### Awareness of chemotherapy-associated ototoxicity

All the oncologists and pharmacists indicated that they were aware that certain cancer chemotherapy drugs may cause hearing loss in comparison to 33% of the nursing staff (*p* = 0.010).

#### Identification of common ototoxic medication

Whilst all audiologists were aware that certain cancer chemotherapy drugs may cause hearing loss, their knowledge regarding other drug classes that may be ototoxic appeared to be limited. This was demonstrated by them correctly identifying medication for TB, that is, aminoglycosides to be ototoxic but not being able to name the drugs. Audiologist B reported:

‘All your mycin drugs, what you call that I forgot the name but it’s that class of drugs, then obviously the cancer chemo drugs and more recently we finding renal patients so it’s your loop diuretics and those kind of things.’ (Audiologist B, male, 7 years experience)

Furthermore, audiologists A and B correctly identified loop diuretics as being ototoxic, whilst audiologists A, B and C did not indicate ARTs to be ototoxic, and audiologist D indicated her uncertainty about ARTs being ototoxic:

‘I’m not sure if the ARVs also cause hearing loss but I also don’t know their names.’ (Audiologist D, female, 18 years experience)

### Patient-reported symptoms

#### Reduced hearing sensitivity

Reduced hearing sensitivity and pain in the ears were two of the most common auditory symptoms reported to oncologists and nurses by cancer patients, whilst noises in the ear and hypersensitivity to sounds were seldom or not reported at all. All audiologists indicated that patients on cancer chemotherapy would generally complain of reduced hearing sensitivity and speech discrimination problems. The hearing loss was described to be permanent and progressive, with audiologist C reporting:

‘Once they’ve presented with a hearing problem after about the second round of chemo, it does get worse by the end of the treatment.’ (Audiologist C, female, 3 years experience)

Audiologist C indicated that the hearing loss generally appears after approximately 6 weeks. Audiologists A and B indicated that the progression of the hearing loss was dose-dependent:

‘I think depending on the dosage and how long they have been on it.’ (Audiologist A, male, 8 years experience)

#### Tinnitus

In addition, audiologists B, C and D also indicated tinnitus, which is permanent; however, audiologist C reported:

‘They generally describe it as a roaring noise and that’s their major issue.’ (Audiologist C, female, 3years experience).

However, Audiologist D described the tinnitus as:

‘… usually high frequency.’ (Audiologist D, female, 18 years experience)

Audiologist B also reported dizziness because of vestibulo-toxicity being a side effect of cancer chemotherapy.

### Awareness of the responsibilities of healthcare personnel

#### Team approach in ototoxicity monitoring

Seven oncologists (100%), seven nurses (78%) and nine pharmacists (69%) considered the audiologist to be part of the team managing a patient with cancer. All audiologists indicated that the hospital did not currently have a team approach for managing adult patients on cancer chemotherapy, with one noting:

‘I don’t think that we are a part of the team if there even is a team.’ (Audiologist, A, male, 8 years experience)

While Audiologist D reported:

‘No there isn’t a team at the hospital with oncology and for cancer patients.’ (Audiologist D, female, 18 years experience)

Furthermore, the audiologists only receive referrals from the oncology department when the patient complains of reduced hearing sensitivity, with Audiologist C, indicating:

‘It usually is whatever the patient reports, then they will refer to that professional as needed.’ (Audiologist C, female, 3 years experience)

#### Pretreatment counselling (information giving)

Two oncologists (29%) and two nurses (22%) indicated that it was the nurse’s responsibility to provide information to patients about the ototoxic effects of medication, whilst all clinic personnel (100%) felt that it was the oncologist’s responsibility to do so. Furthermore, one oncologist (14%) and two nurses (22%) felt that it was the pharmacist’s responsibility to provide patients with information about the possible ototoxic effect of medication, whilst one oncologist (6%) indicated that it was the audiologist’s responsibility to do so. The majority of the pharmacists indicated that it was either their responsibility (85%) or that of the oncologists (92%) to provide patients with information about the possible ototoxic effects of medication, whilst a lesser number indicated that such a role should be delegated to either the nurse (38%) or the audiologist (38%) (Table 4).

Audiologist A reported that it was both the oncologist’s and the audiologist’s responsibility to inform patients about the possible ototoxic effects of chemotherapy:

‘I think the doctors initially do tell them, yes that there is a chance of this happening because they are prescribing the medication because they are responsible for that. And then you need the audiologist to reinforce that.’ (Audiologist A, male, 8 years experience)

However, audiologist C indicated that it is the oncologist’s responsibility to inform the patients about the ototoxic effects of chemotherapy,

‘I think that it is the doctor because it’s not just audiology that’s involved.’ (Audiologist C, female, 3 years experience)

Another also indicated that the audiologist is responsible for setting up the ototoxicity-monitoring programme:

‘…you know any audiology programme needs to be set up by a key player or audiologist.’ (Audiologist B, male, 7 years experience)

#### Referrals to ototoxicity-monitoring programme

With regard to assuming responsibility for the referral of patients at risk for hearing loss to the ototoxicity-monitoring programme, all pharmacists (100%) felt that it was the oncologists’ responsibility to do so. In addition, two pharmacists (15%) reported that it was the nurse’s responsibility to do so, whilst three pharmacists (23%) indicated that it was their responsibility and one pharmacist (8%) indicated that it was the audiologist’s responsibility to do so. Audiologist B indicated:

‘Ideally hopefully the doctor would know that they need to come for hearing….’ (Audiologist B, male, 7 years experience)

Whilst another stated that all patients on cisplatin chemotherapy should be sent for audiological assessments:

‘I think that they all should be sent, as long as they are on the drugs.’ (Audiologist A, male, 8 years experience)

### Ototoxicity-monitoring programme

#### Awareness of ototoxicity-monitoring programme at the hospital

Five oncologists (71%) and one nurse (11%) indicated that their facility does not have an ototoxicity-monitoring programme, whilst two oncologists (29%) and eight nurses (89%) were not sure if one existed. The Fisher’s exact test revealed a significant difference (*p* = 0.021) between the oncologists and oncology nurses’ responses about the ototoxicity-monitoring programme at the hospital. In addition, most of the oncologists indicated that there was no protocol for monitoring patients’ hearing (57%) or was unsure if one existed (43%). Similarly, nurses were unsure if such a protocol existed (78%).

All audiologists indicated that there is no ototoxicity-monitoring programme at the hospital, with audiologist B further stating:

‘No, we don’t have a programme but it’s not to say that we won’t have it.’ (Audiologist B, male, 7 years experience)

#### Elements of an ototoxicity-monitoring programme

Whilst none of the audiologists were able to correctly state all the key elements of an ototoxicity-monitoring programme, they did indicate baseline testing prior to treatment, monitoring tests to enable the earliest detection of the hearing loss and follow-up tests to determine post-treatment hearing status, but were unable to correctly indicate the intervals for these assessments. Audiologists A and B identified pretreatment counselling as an essential element of an ototoxicity-monitoring programme; however, audiologist C indicated:

‘I think it’s important before they go onto chemotherapy that it’s explained the different side effects the treatment could have for them from the doctor’s point of view.’ (Audiologist C, female, 3 years experience)

With regard to the baseline assessment, audiologists B and C correctly indicated the audiological tests to be conducted. In addition, audiologist B indicated that it is the audiologist who should set up and be in charge of such a programme:

‘…[*B*]ut the audiologist should be in charge of a programme like that but we don’t have anyone yet.’ (Audiologist B, male, 7 years experience)

With no ototoxicity-monitoring programme in place, it was not surprising to note that none of the audiologists reported on establishing criteria for determining ototoxicity as an element of the ototoxicity-monitoring programme.

### Current practices in the identification and management of cancer patients at risk for hearing loss at the hospital

#### Identification of at-risk patients

All pharmacists reported that they were consulted less than 10 times in the previous month about drug interactions.

Two pharmacists (15%) indicated that the hospital has a system that allows patients at risk for hearing loss to be identified; however, only one pharmacist indicated alerting the clinical staff to patients at risk for an ototoxic hearing loss. When reduced hearing sensitivity is reported by a patient, nurses indicated that such patients were referred to the Ear-Nose-Throat (ENT) specialist (89%); however, the oncologists either referred to the ENT specialist (43%) or the audiologist (86%). The majority of the oncologists (86%) and nurses (78%) indicated that they provided information to patients on the possible ototoxic effects of medication prior to commencing chemotherapy.

With regard to enquiries about a patient’s medical history and exposure to drugs used to treat specific conditions, all the oncologists enquired about the existence of comorbidities such as TB and HIV. Majority of the nurses (56%), however, only enquired about HIV infection.

#### Referrals to audiology

All audiologists reported that the oncology department does not make many referrals, indicating:

‘I can’t say that oncology has been really a referral base for us.’ (Audiologist D, female, 18 years experience)

‘The oncology department does refer to us sometimes so we do a baseline audio and monitoring as they see fit.’ (Audiologist C, female, 3 years experience)

#### Audiologist’s role

All audiologists indicated that their current role involved conducting audiological assessments. Audiologist B indicated that a baseline is conducted followed by monitoring evaluations at 1 and 6 months post-treatment, respectively. Furthermore, audiologist B indicated that a full diagnostic audiological assessment is undertaken:

‘It’s a full diagnostic audio, so air, bone, reflexes, well your full immittance and speech [*pause*] discrimination.’ (Audiologist B, male, 7 years experience)

However, audiologist A indicated that baseline audiological assessments do not commonly occur:

‘I don’t think much baselines happen.’ (Audiologist A, male, 8 years experience)

In addition, audiologist C indicated that audiological monitoring occurs only after treatment, and the patient is then referred to the base hospital for ongoing monitoring, and further elaborated:

‘Obviously it wouldn’t make sense for the patient to travel on 2 days and come to this hospital when they have one close by.’ (Audiologist C, female, 3 years experience)

Audiologists A and B reported that doctors are informed of the changes in hearing, and patients are subsequently referred to the base hospitals in their area of residence should they present with an ototoxic hearing loss, and/or are fitted with hearing aids. Audiologists A and C also reported that they counsel the affected patients.

## Discussion

With the increasing incidence of cancer in South Africa, affected patients would expect healthcare professionals to be aware of all side effects of the medication prescribed. Results of this study have revealed that all oncologists, pharmacists and audiologists are aware that certain chemotherapy drugs used in cancer treatment may cause hearing loss. This finding adds to the body of published evidence in a similar respect (De Andrade et al., 2009; Wium & Gerber, 2016). However, an area of development has been identified to improve the awareness of nursing staff to the ototoxic nature of some chemotherapy drugs. Knowledge and awareness of this drug attribute is fundamental for informing the patient during pretreatment counselling and/or subsequent visits to ensure the early identification of ototoxicity. Such information serves to give the patient a sense of greater control over their well-being (Pillay, 2002) at a time when their emotional state may be most negatively affected. In spite of the KwaZulu-Natal province recording the highest HIV infection rate in South Africa, only one audiologist mentioned that antiretroviral (ARV) drugs or ART may be ototoxic. As patients being treated for cancer may also be HIV-positive and on ART regimens, this dual comorbidity can present unique challenges to affected patients. Hence, ensuring an adequate enquiry with respect to medical history and knowledge of ototoxic drugs can ensure timeous identification of hearing loss within at-risk populations.

As ototoxic hearing loss is often only detected when a communication problem becomes evident (Fausti, Wilmington, Helt, Helt, & Konrad-Martin, 2005), patients need to be informed of the early symptoms of ototoxicity to ensure its early identification and management. Whilst all audiologists were aware of the symptoms of ototoxicity, majority of the oncologists and nurses reported that patients do not complain of the ‘subtle’ symptoms of cisplatin ototoxicity, such as tinnitus. Given that tinnitus is a very early predictor of ototoxicity, patients ought to become aware of such symptoms to ensure timely notification to healthcare personnel to prevent delayed management of the hearing loss (De Andrade et al., 2009). There is thus a clear role for the audiologist to be involved in the pretreatment counselling of cancer patients receiving chemotherapy. However, not all participant audiologists were aware that pretreatment counselling fell within their domain. In addition, the participants were not clear as to their roles and responsibilities within an ototoxicity-monitoring programme.

Information about the ototoxic effects of medication should be provided by the oncologist, nurses and audiologist, with the audiologist providing more details on the signs and symptoms (American Speech and Hearing Association 1994); however, in the current study, pharmacists have also been identified by healthcare personnel as being responsible for providing patients with such information. Furthermore, recommendations regarding hearing conservation should be provided to the patient by the audiologist (Dabrowski & Hussain-Said, 2010); however, findings of this study revealed that this is not the case.

Whilst doctors have an ethical obligation to inform patients about the side effects of prescribed medication to permit patients to make informed decisions, a recent South African study revealed that a minority of the study participants, that is, doctors, routinely disclosed ototoxic risks to their patients (Wium & Gerber, 2016). This failure to disclose information to patients was attributed to a possible lack of awareness of such risks as well as possible language barriers affecting healthcare professional–patient interaction (Wium & Gerber, 2016). Lack of information to patients provides a barrier for patient care and intervention if and when required (De Andrade et al., 2009).

Whilst majority of the healthcare personnel correctly identified the oncologist as the main custodian for providing patient information on the ototoxic effects of medication, the audiologist was not identified as a key role player in such processes, even by some of the audiologists themselves. This indicates that audiologists may not have viewed themselves as custodians for the provision of such information and to be a part of the larger team. If the audiologists do not see themselves as part of the process, then other healthcare professionals are unlikely to do as well in the collective management of affected patients. Therefore, Wium and Gerber (2016) emphasised that audiologists in South African hospitals should advocate their role in ototoxicity monitoring so as to promote referral of affected patients to an ototoxicity-monitoring programme.

Whilst the pharmacists have identified the oncologists as being responsible for referring patients at risk of hearing loss, they have not clarified their own roles within an ototoxicity-monitoring programme either, namely, alerting oncology staff and audiologists to patients at risk for a hearing loss. It is likely that the awareness of chemotherapy-associated ototoxicity, its related symptoms and the roles and responsibilities of each of the healthcare personnel within such a programme needs to be delineated and conveyed to ensure a cohesive and effective programme. Whilst majority of the oncologists revealed that there is no protocol or programme for monitoring the patient’s hearing status, majority of the nurses were not sure if one existed. This has highlighted the need for further internal communication and knowledge in complex environments.

The study further indicates that information on and awareness of ototoxicity will only serve to enhance early detection and timely management of patients, thus highlighting an additional role for audiologists in the establishment of, and participation in, continuing professional development activities on ototoxicity monitoring to raise awareness and grow the knowledge base for other healthcare professionals (Wium & Gerber, 2016). Such activities have the potential to enhance involvement of personnel from the different relevant specialities and ensure that a successful and robust programme with the following key elements is implemented: (1) utilising specific audiometric criteria for cochleotoxicity, (2) identification of patients, (3) pretreatment counselling regarding the potential effects of the treatment on the auditory system, (4) baseline testing prior to treatment, (5) monitoring tests at intervals suitable to enable the earliest detection of the hearing loss and (6) follow-up tests at intervals suitable to determine post-treatment hearing status (American Speech and Hearing Association 1994). Access to resources such as the comprehensive programme for the monitoring of cochleotoxicity by the American Speech and Hearing Association (1994), as well as the recently released ototoxicity-monitoring guidelines by the Health Professions Council of South Africa (2018) coupled with suitable discussion forums can assist the audiologist in setting up an ototoxicity-monitoring programme, employing a multidisciplinary team approach.

This team approach would not be successful without effective communication and cooperation between audiologists, oncologists, nurses and hospital pharmacists. The findings of this study highlight that the pharmacists at the study site are not fully utilised, as they are consulted less than 10 times a month about drug interactions. A robust hospital system for identifying patients at risk for hearing loss would be an important development through the use of an automated database, which would allow the audiologist to identify those individuals receiving cochleotoxic treatment and to target these patients via automated referral generation (American Academy of Audiology, 2009). This referral system can involve the expertise of pharmacists who have access to the patient’s list of medication.

It was evident that whilst the oncologists enquired about common illnesses such as TB, HIV, pain and fever, there was limited awareness around the ototoxic effects of quinine with only one oncologist enquiring about it. This can be attributed to the numerous reports of medication used in the treatment of TB, HIV, malaria, and pain and fever being ototoxic (Campbell, 2000). Furthermore, majority of the nurses did not enquire about these conditions and as such, patients who contract these illnesses during the course of chemotherapy may not report them, as they are generally not seen by the oncologist during each cycle of chemotherapy but rather by the nurses. Oncologists and nurses also did not display awareness of patients being genetically susceptible to hearing loss as only one oncologist and three nurses reported enquiring about family history of hearing loss. It is likely that the nurses do not enquire about family history of hearing loss, as well as previous medical history as they assume that this has been addressed by the oncologists and that there is no change since the last enquiry.

Patients complaining of reduced hearing sensitivity were more likely to be referred by the nurse to the ENT specialist, as opposed to the audiologist, whilst majority of the oncologists reported referring the patient to the audiologist. The findings of this study do not concur with the literature where it was reported that the participant oncologists had superficial knowledge of the role of the audiologist (De Andrade et al., 2009). In our study, majority of the oncologists indicated referring patients complaining of reduced hearing sensitivity to the audiologist. This is in keeping with the reports of the audiologists who indicated that patients are only referred to them once there are complaints of reduced hearing sensitivity; hence, conducting baseline assessments is not common practice.

All audiologists displayed awareness of their role of conducting audiological assessments in an ototoxicity-monitoring programme; however, this awareness does not translate to practice, as there was disparity in their responses regarding the time frames and tests currently being adhered to at the hospital, with one audiologist reporting that ‘audiological monitoring occurs only once, after the chemotherapy treatment’. Furthermore, not all audiologists displayed awareness of their role in managing hearing loss, as only two audiologists reported informing doctors of the change in the hearing, counselling and fitting the patient with hearing aids. Whilst the reasons for not practising ototoxicity monitoring were not explored in the current study, the researchers are of the view that the lack of a standardised comprehensive national ototoxicity-monitoring protocol at the time of data collection may have been a contributing factor in addition to limited human resources (Coovadia, Jewkes, Barron, Sanders, & McIntyre, 2009). South Africa has a low audiologist to population ratio with 1:100 000 compared to 4:100 000 in the United Kingdom (Fagan & Jacobs, 2009). This may result in audiologists not fully engaging with ototoxicity monitoring. Similarly, the nursing staff capacity may also impact their levels of patient engagement. Pillay (2002) indicated that nurses in the South African public health sector were highly dissatisfied with their pay, workload and the resources available to them and the further recommendation of an ototoxicity-monitoring programme can be impacted by such factors.

Whilst this study was conducted at a single institution and a larger multisite study to assess awareness and practice within the broader South African context will be extremely valuable, the results do highlight areas for improvement in the practice and care of affected patients. Similar resource-limited environments may experience similar variation with respect to ototoxicity knowledge, hence influencing the care of affected patients.

## Conclusion

It is evident that there is awareness amongst healthcare personnel that certain chemotherapy drugs, such as cisplatin, can cause hearing loss. However, awareness of the roles and responsibilities of healthcare personnel as well as the success of a monitoring programme all dwell upon an effective and cohesive multidisciplinary team where the roles and responsibilities are delineated for proper referral, counselling, identification and appropriate management. There is also room for improvement with respect to increasing awareness amongst audiologists around the elements of ototoxicity monitoring.
